# Treatment of Diseased Gums

**Published:** 1869-06

**Authors:** 


Treatment of Diseased Gums.—A writer in the *London Lancet* recommends the following treatment of diseased gums: The teeth should be washed night and morning with a moderately small and soft brush; after the morning ablution, pour on a second toothbrush, slightly damped, a little of the following lotion, and apply it to the affected parts: Carbolic acid, one scruple; rectified spirits of wine, two drachms; distilled water, six ounces. By the use of this preparation, suppurative action is kept under, and the gums get firmer and less tender.—*The Medical Record.*

The anatomical museum of the St. Louis Medical College was destroyed by fire recently. It was the richest and most valuable of its kind in that section of the country. No insurance on it. We presume the loss included Dr. Pope's extensive and valuable private collection.

