# Correction

**Published:** 1869-08

**Authors:** 


### CORRECTION.

THE notice copied into the REGISTER of June, on page 259, of the burning of the Anatomical Museum of the St. Louis Medical College, is, as we are informed by the Dean of that institution, a mistake. He says, "the building burned was the O'Fallon Dispensary and the Museum of the St. Louis Academy of Sciences." We copied the notice from an exchange, presuming it was correct. T.

